# Supplementary material for: A UMLS-based spell checker for natural language processing in vaccine safety
Source: BMC Med Inform Decis Mak. 2007 Feb 12;7:3. doi: 10.1186/1472-6947-7-3 (PMC1805499; doi:10.1186/1472-6947-7-3)
Supplement: Additional file 13 — RAP application directory. Sets up directory for RAP (RDF application for PHP) source code [file 1472-6947-7-3-S13.gz › rap/doc/phpdoc/__filesource/fsource_ontModel__ontModelOntVocabulary.php.html]

xml version="1.0" encoding="iso-8859-1"?


File Source for OntVocabulary.php


# Source for file OntVocabulary.php

Documentation is available at OntVocabulary.php

```
1. <?PHP
2. /**
3. * ----------------------------------------------------------------------------------
4. * Class: OntVocabulary
5. * ----------------------------------------------------------------------------------
6. *
7. * @package     ontModel
8. */

10. /**
11. * Base Class that encapsulates the elements of a general vocabulary
12. * corresponding to a particular ontology language. The intent is that,
13. * using a given vocabulary, a given RDF model can be processed as an
14. * ontology description, without binding knowledge of the vocabulary
15. * into this Class
16. *
17. * <BR><BR>History:
18. * <LI>10-07-2004                : First version of this class.</LI>
19. *
20. * @version  V0.9.1
21. * @author Daniel Westphal <mail at d-westphal dot de>
22. *
23. *
24. * @package     ontModel
25. * @access    public
26. ***/
27. class OntVocabulary
28. {

30. }
31. ?>
```

Documentation generated on Thu, 7 Jul 2005 13:42:34 +0200 by phpDocumentor 1.3.0RC3
